# Note-taking fosters distance video learning: smartphones as risk and intellectual values as protective factors

**DOI:** 10.1038/s41598-024-67898-7

**Published:** 2024-07-23

**Authors:** Markus H. Hefter

**Affiliations:** Department of Psychology, Bielefeld University, 33615 Bielefeld, Germany

**Keywords:** Human behaviour, Information technology

## Abstract

Distance video learning, especially with how-to videos, has become extremely popular. Whereas previous studies revealed note-taking as a prominent and promising support measure for video learning in the lab, we focus on note-taking while learning from a how-to video in a distance learning setting. Meanwhile, learners’ digital distraction and smartphone usage have become widespread and excessive, potentially harming learning. We thus also aimed to analyze potential risk and protective factors associated with learning with how-to videos, such as smartphone usage time and intellectual values. We conducted an online field experiment involving 59 psychology students, who learned with a short how-to video on plumbing. We found that note-takers outperformed non-note-takers in a posttest about the video content. Furthermore, this note-taking effect on learning outcomes was mediated by learning engagement. Besides note-taking and intellectual values as contributing positively to learning outcomes, we also identified the mean daily smartphone usage time as a risk factor to learning. Overall, our results show how beneficial it is for students to take notes while learning with how-to videos in a distance learning setting. Conversely, learners should avoid digital distractions, in particular through smartphones.

## Introduction

Video-based learning has been on the rise for decades in various forms, be it educational or documentary shows on TV, video lectures for online courses, or video demonstrations^1,2^. In this paper, we focus on video demonstrations, which are videos that demonstrate how to solve a certain task. They are therefore often called video examples or simply how-to videos. Such how-to videos have become extremely popular on YouTube^3^. Already in 2015, searches related to “how to” on YouTube were growing 70% year to year^4^. Such growing popularity of how-to videos has naturally influenced research on effective learning with how-to videos. Various effective learning environments relying on how-to videos have recently emerged. Examples of such video-based learning environments cover diverse topics such as how to be creative^5^, how to assemble circuits^6^, how to generate arguments^7^, or how to diagnose car malfunctions^8^.

In light of how-to videos’ popularity, researchers have been seeking how to optimize learning from such how-to videos. We focus on a very prominent and promising approach: *Note-taking*. Whereas previous studies focused on note-taking as a support measure for video learning in the lab, we focus on note-taking while learning from a video in a distance learning setting. However, in such a distance learning setting, learners are even more prone to digital distractions than in on-site learning settings. Hence, when thinking about note-taking to support learning from online videos, it is feasible to also consider the detrimental effects of digital distraction and what might risk and protect learning with online how-to videos. In the present paper, we have therefore aimed to analyze potential risk and protective factors, such as smartphone usage time and intellectual values.

### Note-taking supports learning from videos—in the lab

Piolat and colleagues^9^ understand notes as “short condensations of a source material that are generated by writing them down while simultaneously listening, studying, or observing” (p. 292). Generating condensations is an apt description of note-taking because it makes clear that note-taking is more than simply transcribing heard or read information. Rather, note-taking is a complex and thus demanding cognitive activity. It comprises various processes such as selecting and comprehending information as well as reformatting it (e.g., abbreviating and paraphrasing) and writing it down^9^. All these processes demand central executive cognitive resources. In other words, note-taking requires not only active listening but also active cognitive processing.

Said cognitive processing gained the major theoretical and practical interest as the main function of note-taking. Peper and Mayer^10^ called it the *encoding function*. The other function of note-taking, namely external storage for later review, is *not* this paper’s focus. Decades ago, in a series of vintage experiments, Peper and Mayer^10,11^ and Shrager and Mayer^12^ let students learn with various how-to videos in a lab and compared note-takers with non-note-takers. In their experiments, they simply instructed learners in the note-taking groups to take notes on a given sheet of paper. By contrast, learners in the non-note-taking groups were told to merely watch the video closely. Peper and Mayer^10^ found that writing down notes while watching a 16-min video on computer programming or a 22-min video on statistics was beneficial to students in a subsequent test. Similar results were achieved in studies involving a 23-min video about how a car engine works^11^ and a study involving a 11-min video on how to use a camera^12^.

More recent studies achieved similar results. For instance, in one of their experiments with 77 students, Bohay and colleagues^13^ showed how note-taking was beneficial when learning with three 9-min video lectures. Furthermore, Wong and Lim^14^ conducted two lab experiments with 200 learners watching two 9-min video lectures. They instructed their learners in the note-taking group to write their notes sown on a sheet of paper while watching the videos. They found that note-takers outperformed non-note-takers (and even photo-takers) in a subsequent posttest. Finally, Mueller and Oppenheimer found that taking notes longhand tends to favor cognitive processing more than typing notes on a laptop^15^: When taking notes longhand, learners are inclined to synthesize and summarize information rather than merely type verbatim notes.

Condensing all these findings, note-taking has the potential to be a *generative activity*^16^ that results in students connecting new information with prior knowledge. In other words, note-taking can lead to cognitive processing that helps students to better understand and remember video content. The aforementioned studies were conducted under lab conditions, however. Time and place were fixed, and a supervisor was present.

### Supporting learning from videos—in a distance learning setting

It is still an open question whether the aforementioned beneficial effects of note-taking on learning from videos not only occur in the lab, but also in a distance learning setting. Distance video learning is an asynchronous scenario pushing the boundaries of a fixed time, place, and device. Furthermore, the lack of supervision could open the door for digital distraction—an issue discussed later in this paper.

There are indeed studies addressing distance video learning and how to enhance it with prompts to let students deeply process the videos. For instance, McClellan and colleagues^17^ enhanced a 30-min video lecture on physics with cognitive, metacognitive, or no prompts. Cognitive prompts aimed to foster cognitive learning strategies such as organizing and elaborating, whereas metacognitive prompts aimed to foster metacognitive knowledge and self-regulation. Their results show that the cognitive prompts were beneficial, as those students who received them outperformed their non-prompt and metacognitive fellows in a posttest. As another example, Hefter and Berthold^7^ enhanced videos on argumentation skills with either so-called self-explanation or notes prompts. In a similar study^18^, a video lecture on psychology was enhanced with various prompts (notes, self-explanations, and elaborations). Briefly put, the prompt types revealed their beneficial effects on learning processes, but not on learning outcomes. Both studies^7,18^ underscored that it is not so much the prompt itself, but the cognitive process while watching the video that predicts learning outcomes.

However, these studies do not answer our open question for mainly two reasons: a) they made no direct comparison between note-taking versus non-note-taking and b) they took the segmenting approach. The segmenting approach, recommended by Fiorella and Mayer^19^, means presenting the video in segments. In a typical study such as the three mentioned above^7,17,18^, each video segment is followed by a prompt with a text box in which to type in text. The time given to answer a prompt is usually unlimited. The next video segment does not start until the learners have submitted a response to the prompt. However, this segmenting approach takes away the temporal pressure of simultaneously maintaining the mental representation of the spoken information to grasp and condense it into a note while receiving continuous updates of this spoken information. Furthermore, how-to videos on the internet are usually not presented in segments followed by prompts and text boxes.

Overall, these studies did not focus on comparing note-taking to non-note-taking while simultaneously watching a video in a distance learning setting. By contrast, this is precisely our focus in the present study. To ensure high ecological validity, we induced no supervision or impression thereof, such as text boxes. Instead, we transferred Peper, Shrager, and Mayer’s^10–12^ vintage experimental approach from the lab into a distance learning setting and simply asked learners to take notes on a sheet of paper.

### Risks of digital distractions

Stepping away from the aforementioned supervised lab condition to an unsupervised setting bears the risk of digital distractions known as digital off-task behavior. A plethora of studies has already highlighted the detrimental role of digital off-task behavior on learning, namely test performance, recall, reading comprehension, note-taking, self-regulation, and efficiency^20–24^. The reason digital distraction deters learning is straightforward and almost a truism: human working memory only has limited cognitive resources. When these resources are exploited by off-task behavior, they become unavailable for deep processing the learning material^25,26^. Obviously, reducing digital distraction is therefore strongly recommended for (effective) learning. But actually reducing digital distraction seems almost impossible.

As outlined below, students have generally been very prone to digital distraction for many years. Already over a decade ago, Burak^27^ asked 774 American university students (*M*_*age*_ = 20.75 years) and over 50% of them admitted to engaging in texting—while sitting in class. When McCoy^28^ asked over 700 American university students, they admitted using their digital device over 10 times for off-task purposes during a typical school day. Some years later, the follow-up survey^29^ involved over 1000 American and Canadian college students who spent more than 19% of their time using a digital device for non-class purposes. In 2021, a study with 125 Swiss university students revealed similar numbers^30^: Students spent over 19% of their time using a digital device for off-task purposes during a typical 90-minute university lecture. These numbers refer to behavior while actually sitting in class under the eyes of a lecturer or teacher.

The numbers in unsupervised settings, such as asynchronous distance learning are even higher: even back in 2012, when Burak asked American university students about their behavior during online courses (which 333 of them attended), more students (~ 70%) claimed to have engaged in texting than did while sitting in class (~ 50%)^27^. In a recent survey^31^ with 630 Romanian university students, 95% admitted checking their devices in online courses for off-task purposes, compared to 75% in face-to-face courses. Another recent study^32^ with 690 Swiss psychology students in six hybrid classes revealed that online learners are more susceptible to distraction than on-site learners. This seems plausible as online learning is not only unsupervised, it also relies on digital devices, which themselves tempt learners into off-task behavior (such as social networks, texting, shopping, etc.)^32^

Summing up, students tend to carry a high risk of engaging in digital off-task behavior, especially during distance learning. Learners are barely able to withstand the enormously addictive potential of their digital devices, especially smartphones.

### Mean daily smartphone usage time as a risk factor for distance video learning

In this paper, we focus on smartphones as the primary source for digital distraction, for two reasons, namely enormous prevalence and excessive usage. In prevalence terms, smartphones have become ubiquitous by now. The percentage of Americans aged between 18 and 29 who own a smartphone has risen from 52% in 2011, 79% in 2013, 86% in 2015, to 96% in 2021^33,34^. The numbers in Germany are similarly high. For example, in 2022 86% of students aged between 10 and 12, 95% of those aged 13 to 15, and 96% between 16 and 18 owned a smartphone^35^.

Concerning excessive usage time, a plethora of studies revealed associations between excessive smartphone usage (also called smartphone addiction or problematic smartphone usage) and a myriad of negative aspects in various fields^36–39^. These negative aspects include lower sleep quality, attention deficits, depressive and anxiety symptoms, reduced physical fitness, shyness, and low self-esteem. Nevertheless, students and even children have become excessively reliant on their smartphone and seem almost permanently attached to them. For instance, an observational study in Germany with a sample of ~1400 students aged between 10 and 19 revealed that about 10.5% (15.3% when alone) used their smartphone while crossing the street, potentially risking their lives^40^. Back in the classroom, a study that assessed smartphone log data from 84 Korean first-year college students found that students spent over 25% of their time operating their smartphones—while actually sitting in class^41^!

When looking at the daily amount of time users spent operating their smartphones, the studies below reported amounts totaling roughly 4 hours a day. Back then in 2015, Saito and Aragaki^42^ collected screenshots from Android smartphones of 113 high school students, revealing a mean daily smartphone usage of 242 minutes. Ellis and colleagues^43^ read out log data from the pre-installed iOS app ‘screen time’ and found that their 238 participants (*M*_*age*_ = 31.88) spent an average of 233 min a day operating their smartphones.

Ohme and colleagues^44^ relied on 47 (out of 404) Dutch-speaking iPhone users (*M*_*age*_ = 40) who agreed to send screenshots of their iOS app ‘screen time’ in the year 2019. The app data showed an average of 257 minutes a day, which was significantly higher than their 146 self-reported minutes. Obviously—although it has been common practice to assess them—self-reported smartphone usage times are not the method of choice. When trying to estimate one’s daily smartphone usage time, it becomes obvious how difficult, if not impossible, it is. The risks of cognitive, social, and communicative biases^45^ are just too much to handle for our already poor time estimation capability^46^.

In 2020, Hefter^47^ employed a sort of “guided data donation method.” This method does not rely on learners’ subjective estimations, not on installing tracking apps on learners’ devices, not on potentially difficult and/or intrusive screenshotting procedures, and not on restricting the sample to either iOS or Android users. Instead, it guided participants through their preinstalled smartphone apps ‘screen time,’ ‘digital balance,’ or ‘digital wellbeing’ to read out their mean daily smartphone usage time. Forty-one (out of 53; *M*_*age*_ = 21.81 years) German university students submitted their data: 235 minutes. Finally, Wu-Ouyang and Chan^48^ used a similar method with 777 Chinese online panel participants (*M*_*age*_ = 26.03) and assessed even higher numbers: 437 minutes.

Summing up, learners operate their smartphones at least 4 hours a day, even when crossing a street and even while attending class. This is why in the present paper we are focusing on smartphones as the primary source for digital distraction during distance video learning and therefore propose the mean daily smartphone usage time as a potential risk factor for learning with videos.

### Intellectual values as a protective factor for distance video learning

Previous research^47^ identified a negative moderate correlation between daily smartphone usage and subjective mental effort spent in an asynchronous video-based learning environment. This correlation might show that learners prone to excessive smartphone usage are less willing to invest mental effort during distance learning. This correlation can also be discussed in light of a modern form of cognitive miserliness^49^. Learners less willing to invest mental effort during distance learning might also be more prone to relying on outsourcing information and cognitive processes on their smartphone.

Against this background, excessive smartphone usage might go hand in hand with the tendency to be unwilling to invest cognitive cost. We thus aimed to find a protective factor that reduces this risk factor’s impact. Such a protective factor would be a learner’s attitude or characteristic that is obviously positively associated with intellectual engagement. *Intellectual Values* might constitute that protective factor and play a major role in how committed learners engage in deep processing and thus learning from a video. Kuhn and Park^50^ propose intellectual values as representing the “perceived value of intellectual activity to a cultural group” (p. 115). We can describe it as the amount an individual considers intellectual engagement as intrinsically worthwhile. In other words, “intellectual engagement, [is] not simply [considered] as a means to personal advancement but as an intrinsically valuable activity in its own right (p. 123). Kuhn and Park mention the similarity to a construct such as *Need for Cognition*^51^. However, need for cognition is more of an individual attribute and concerned with fulfilling an individual’s goals and needs. Intellectual values, by contrast, are more of a group entity, advancing a group or society as a whole.

Hence, we also aimed in this study to analyze the potential positive influence of intellectual values on learning outcomes. If the mean daily smartphone usage time is a possible risk factor for distance video learning, can intellectual values serve as a protective factor?

### The present study

In the present study, we offered students a short how-to video on plumbing. In a web-experiment, we aimed to analyze the effectiveness of note-taking on learning engagement and outcomes. We also analyzed whether and how smartphone usage time and intellectual values contribute to learning.

First, we assumed that our how-to video is effective. To evaluate its effectiveness, we relied on the concept of self-efficacy, which is based on the work of Bandura^52^. It roughly refers to learners’ confidence in their ability to perform a certain task successfully, and it can predict performance^53^. We assumed an effective how-to video will foster its learners’ self-efficacy. Hence, as a simple effectiveness check, we assumed…

- Hypothesis 1: …our short how-to video on plumbing increases the learners’ self-efficacy on that certain topic.
  Furthermore, considering the aforementioned background, note-taking should benefit learning because it fosters active processing of the learning material. Hence, we assumed…
- Hypothesis 2a: …note-taking fosters learning engagement.
- Hypothesis 2b: …note-taking fosters learning outcomes.
- Hypothesis 2c: …the note-taking effect on learning outcomes is mediated by learning engagement.
  Referring to additional predictors (i.e., risk and protective factors) of learning outcomes, we assumed that…
- Hypothesis 3a: …Smartphone usage contributes negatively to learning outcomes.
- Hypothesis 3b: …Intellectual values contribute positively to learning outcomes.

## Method

### Sample and design

An a priori power analysis (with G*Power 3.1.9.2)^54^ for a one-tailed *t* test (*d* = 0.65 [medium to large effect], α err prob = .05, power [1-β err prob] = .80) revealed a required sample size of 60 (30 participants per group). We recruited 60 university students to take part in this study during the winter semester 2023-2024. They were undergraduate psychology students, who had enrolled in the university’s platform for research participants to fulfill part of their research participation requirements without receiving additional compensation. We had to exclude one participant who did not finish the study. The final sample comprised *N* = 59 (36 female, 18 male, 5 n/a; *M*_*age*_ = 24.51 years, *SD* = 5.80).

All participants were randomly assigned to one of two experimental conditions: (A) video with a note-taking request (*n* = 29), (B) video with no note-taking request (*n* = 30). Moreover, we asked the participants afterwards, whether they actually took notes. Their answers imply a different scenario, namely that the actual note-taking group was not identical to the group given a note-taking request. In other words, not everybody who was told to take notes reported having done so. For this paper, we therefore compared actual note-takers to non-note-takers rather than comparing note-request-recipients to non-note-request recipients. See Table 1 for a cross tab.

**Table 1:** Participants and note-takers

	Condition		
	Note-taking request	No note-taking request	Overall
Note-takers	23	1	24
Non-note-takers	6	29	35
Overall	29	30	59

### Ethical approval

The study was conducted in accordance with the Ethical Guidelines of the German Association of Psychologists (DGPs). It was approved by the Ethics Committee of Bielefeld University (No. 2023–317). All participants gave informed consent.

### Online learning environment and procedure

The participants accessed the online learning environment via a link and were informed that a computer with headphones or speakers is required. The learning environment started with thanks, greetings, mandatory data protection information, and requiring informed consent. Both experimental conditions featured the identical 12-min video demonstration on how to fix a blocked toilet. In this video, a plumber demonstrates different tools in front of a fixed camera. Furthermore, he verbally explains basic information about home sewer systems. The only difference between the experimental conditions was the following: Before the video started, the experimental condition participants were asked to get a pen and a paper to take notes while watching the upcoming video. By contrast, control groups participants were asked to not use any tools such as notes, the internet, etc. Hence, the experimental manipulation closely resembled the classic studies discussed above^10–12^. Participants had video control buttons (i.e., play/pause, volume). After the video, participants were told to put away any notes before they received the questionnaires on learning engagement, interruptions, intellectual values and smartphone usage. They then were given the test on learning outcomes after another reminder not to use any tools such as notes, the internet, etc. Finally, they received a demographic questionnaire.

### Instruments

#### Learning time

The online learning environment logged time stamps so that we could calculate the time participants spent watching the video and potentially taking notes.

#### Learning engagement

We assessed learning engagement with the self-report scale established by Daumiller and colleagues^55^. These authors formed their scale as “selective and face valid single items from established scales” (p. 6). An example item is “I made a lot of effort to understand everything”^56^. In previous studies the scale correlated highly with actual objective learning processes such as the quality of typed notes^57^ and objective learning outcomes, such as posttest performance^58^. We used the mean of all five items as a measure of learning engagement (Cronbach’s α = 0.77).

#### Interruptions

We used one single item^47^: “Were you interrupted by other people or events/incidents during the video?” Participants answered on 5-point scale from 0 (*no interruption*) to 4 (*more than three interruptions*). This item proved to be a quite valid indicator of interruptions that negatively affected learning and posttest performance in previous studies^18,47^.

#### Intellectual values

We relied on the scale of 14 items developed by Hefter and colleagues^59^. Each item described a fictive person who considers an aspect of intellectual engagement worthwhile. These aspects referred to different kinds of intellectual engagement and epistemic behavior^50,60^. They included the valuation of discussions, of systematic observations or experiments, of rational thinking, and of consultation. Participants had to rate subjectively their similarity (“How similar is this person to you?”) to these 14 fictive persons on a Likert-type scale from 1 (*not like me at all*) to 6 (*very much like me*). We used the mean of all 14 items as measure of intellectual values (Cronbach’s α = 0.91).

#### Smartphone usage

To assess mean daily smartphone usage time, we relied on the guided log data report by Hefter^47^. This method asked participants to fetch their smartphones and use preinstalled logging apps, such as iOS’s ‘screen time’ or Android’s ‘digital wellbeing’ or ‘digital balance’. Screenshots helped the participants in guiding them through their smartphone apps according to their operating system. Overall, 55 out of 59 participants (93%) provided their data.

#### Learning outcomes

We presented 16 statements referring to plumbers’ knowledge about blocked toilets, such as ﻿“Too much toilet paper is one of main causes of a blocked toilet.”﻿ We relied on previous research employing confidence weighted true–false items for learning outcomes^61^. This approach reduces the risk of participants simply guessing and allows to differ between misconceptions and missing concepts^62^. Participants should not only to decide between ﻿“I agree”﻿ or ﻿“I disagree”, but also indicate their subjective certainty on a Likert-type scale from 0 (*very uncertain*) to 4 (*very certain*). We multiplied the value for correctness (i.e., + 1 or − 1) by the subjective certainty rating (0 to 4) for each item. As a measure of learning outcomes, we used the mean for all items (Cronbach’s α = 0.80).

#### Self-efficacy

We assessed self-efficacy focusing on the video’s topic (i.e., how to fix a blocked toilet) as pretest and posttest. The stem “Please mark how confident are you about the following…” was followed by four items (e.g., “I can fix a blocked toilet.”). All four items were answered on a Likert-type scale ranging from 1 (*lowest*) to 5 (*highest*). We used the mean of all four items as measure of interest (Cronbach’s α_Pretest_ = 0.81 and Cronbach’s α_Posttest_ = 0.85).

## Results

We took the classic standard approach: 0.5 alpha-level for all tests, η_part_^2^ as the effect size for *F* tests, Cohen’s *d* as the effect size for *t* tests, and Pearson’s correlation coefficient *r* for correlations. For η_part_^2^, values below 0.06 were qualified as small, between 0.06 and 0.13 as medium, and above 0.13 as large effects^63^. For *d*, values around 0.20 were qualified as small, around 0.50 as medium, and above 0.80 as large effects^63^. For *r*, values around 0.10 were qualified as small, around 0.30 as moderate, and above 0.50 as large correlations^63^. Table 2 provides descriptive statistics and correlations for all measures.

**Table 2:** Descriptive statistics and correlations for all measures note

	Means (standard deviations)			Manifest correlations						
	Note-takers	Non-note-takers	Overall	1	2	3	4	5	6	7
Learning time^1^	13.85 (2.25)	12.11 (2.61)	12.82 (2.60)							
Learning engagement^2^	5.39 (1.43)	4.58 (1.23)	4.91 (1.36)	0.18						
Interruptions^3^	0.13 (0.34)	0.34 (0.64)	0.25 (0.54)	− 0.13	− 0.22					
Intellectual values^4^	4.51 (0.75)	4.20 (0.86)	4.32 (0.82)	**0.27**	0.11	** − 0.35**				
Smartphone usage^1^	206.47 (112.84)	266.98 (140.70)	241.68 (132.14)	** − 0.32**	− 0.13	**0.36**	− 0.18			
Prior self-efficacy^5^	2.85 (0.95)	2.60 (0.82)	2.70 (0.88)	− 0.09	**0.29**	0.15	0.06	0.01		
Posttest self-efficacy^5^	3.94 (0.85)	3.36 (0.80)	3.59 (0.86)	− 0.01	**0.53**	− 0.12	**0.30**	− 0.04	**0.59**	
Learning outcomes^6^	2.01 (0.81)	1.38 (0.94)	1.64 (0.94)	**0.33**	**0.39**	− 0.25	**0.48**	** − 0.38**	0.21	**0.41**

^1^Time in minutes.

^2^Scale from 1 (lowest) to 8 (highest).

^3^Scale from 0 (no interruption) to 4 (more than three interruptions).

^4^Scale from 1 (lowest) to 6 (highest).

^5^Scale from 1 (lowest) to 5 (highest).

^6^Scale from − 4 (lowest) to 4 (highest).

Bold correlations: *p* < 0.05 (two-sided).

### Control variables

We observed no statistically significant differences between note-takers and non-note-takers with respect to interruptions, *t*(57) = −﻿ ﻿1.53, *p* = 0.132, *d* = 0.41, intellectual values, *t*(57) = 1.41, *p* = 0.163, *d* = 0.38, smartphone usage, *t*(53) = −﻿ 1.70, *p* = 0.094, *d* = 0.47, and prior self-efficacy, *t*(57) = 1.09, *p* = 0.279, *d* = 0.29. There was, however, a statistically significant effect of note-taking on learning time, *t*(57) = 2.65, *p* = 0.005, *d* = 0.70 (one-tailed *t* test, medium effect). This is no surprise, as note-taking obviously takes time.

### Effect of the video on self-efficacy

In Hypothesis 1, we assumed our how-to video on plumbing would increase the learners’ self-efficacy on that topic. We conducted a repeated measures ANOVA with prior and posttest self-efficacy as the repeated measure and note-taking as the independent variable. After having watched the video, learners reported greater self-efficacy than before regardless of note-taking, revealed by a statistically significant repeated measures main effect *F*(1, 57) = 24.11, *p* < 0.001, η_part_^2^ = 0.58 (large effect). We also detected a main effect of note-taking reflecting note-takers greater self-efficacy, *F*(1, 57) = 4.35, *p* = 0.041, η_part_^2^ = 0.07 (medium effect). There was however no statistically significant interaction effect, *F*(1, 57) = 2.48, *p* = 0.121, η_part_^2^ = 0.04.

### Effect of note-taking on learning engagement and outcomes

According to Hypothesis 2a, note-taking should foster learning engagement. Indeed, the-note-takers reported deeper learning engagement than the non-note-takers, *t*(57) = 2.34, *p* = 0.011, *d* = 0.62 (one-tailed *t* test, medium effect). According to Hypothesis 2b, note-taking should foster learning outcomes. We found that note-takers outperformed the non-note-takers in the posttest, *t*(57) = 2.65, *p* = 0.005, *d* = 0.70 (one-tailed *t* test, medium effect). When comparing only the raw correctness scores, a statistically significant difference between note-takers and non-note-takers also appears, *t*(57) = 1.71, *p* = 0.046, *d* = 0.45 (one-tailed *t* test, medium effect).

### Learning engagement mediates the note-taking on learning outcomes

**Figure 1:**
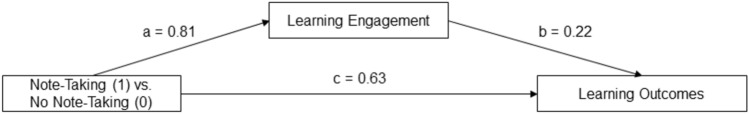
Statistically significant mediation results.

In Hypothesis 2c, we assumed that the note-taking effect on learning outcomes would be mediated by learning engagement. To test this hypothesis, we conducted a mediation analysis via the SPSS macro PROCESS^64^. The independent variable was (reported) note-taking behavior (i.e., note-taking vs. non-note-taking), the dependent variable was learning outcomes, and the potential mediating variable was learning engagement. The PROCESS macro calculated 95% bootstrap percentile confidence intervals from 10,000 bootstrap samples. We noted a significant positive direct effect of note-taking on learning engagement (a path), *B* = 0.81, *p* = 0.023 and a significant positive direct effect of learning engagement on learning outcomes (b path), *B* = 0.22, *p* = 0.013. Finally, we found that note-taking had an indirect effect on learning outcomes via learning engagement (ab path), *B* = 0.18 [0.0047, 0.4638]. The confidence interval, which did not include zero, supports the conclusion that learning engagement mediated the note-taking effect on learning outcomes. The direct effect of note-taking on learning outcomes (c' path) amounted to *B* = 0.45, *p* = 0.064 and the total effect (c path) to *B* = 0.63, *p* = 0.011. Figure 1 shows the mediation results.

### Intellectual values and smartphone-usage predicting learning outcomes

**Figure 2:**
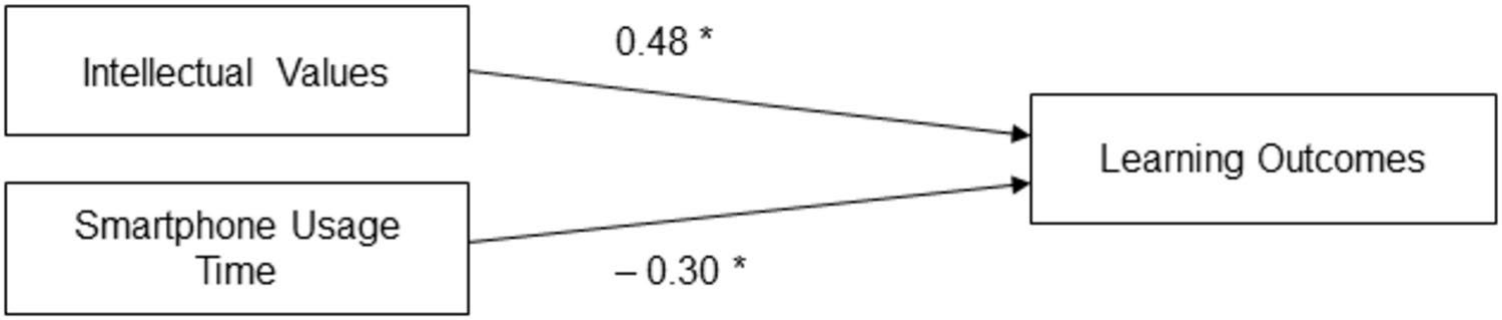
Regression on learning outcomes. Standardized beta coefficients, *p < 0.05.

**Figure 3:**
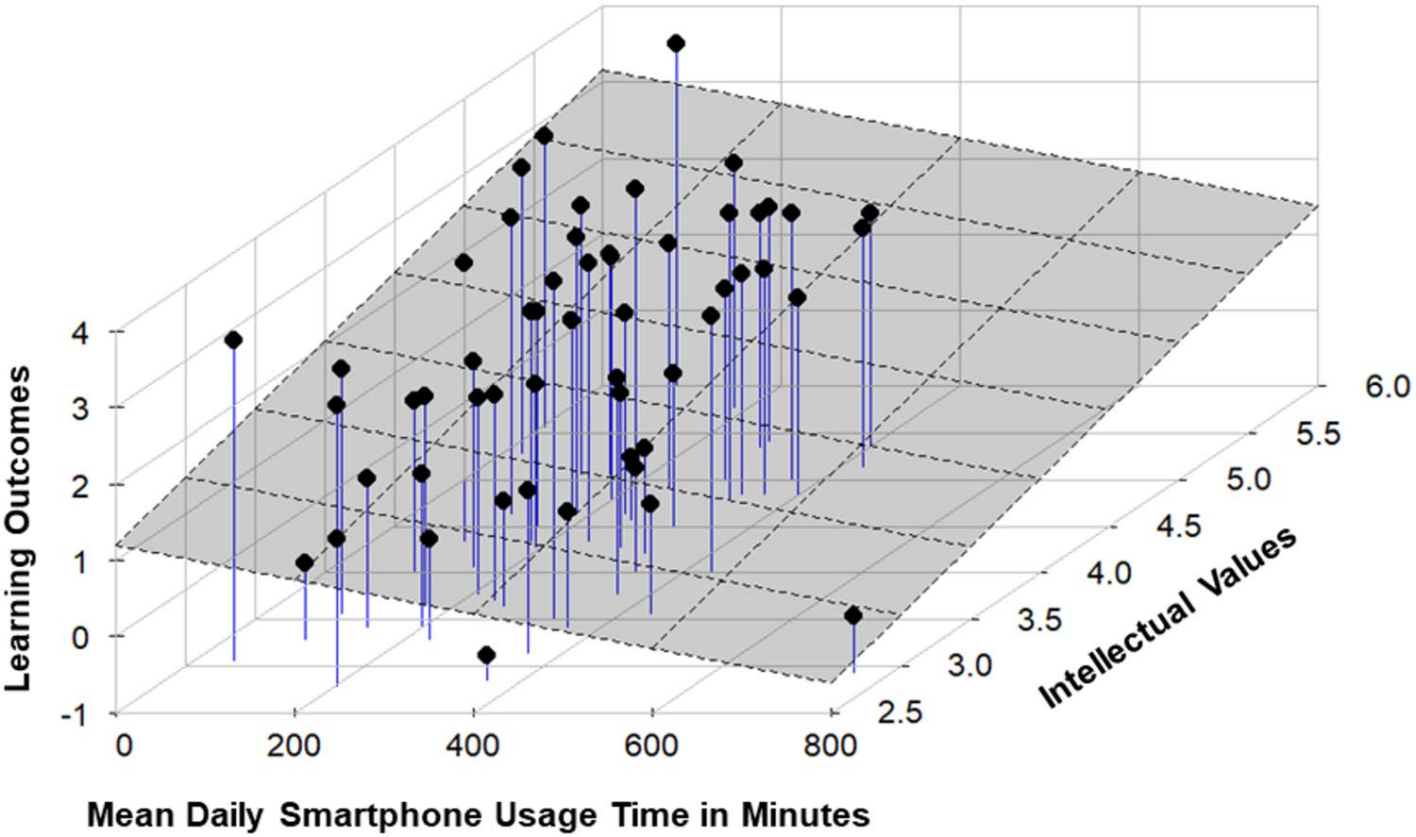
3D scatterplot with regression plane predicting learning outcomes on intellectual values and mean daily smartphone usage time.

As we were interested in whether and how intellectual values and smartphone usage might predict learning outcomes, we carried out a linear regression analysis. The dependent variable was posttest performance. Intellectual values and smartphone usage time were entered as potential predictors. The regression analysis yielded a statistically significant overall result, *F*(2, 54) = 15.45, *p* < 0.001, *R*2 = 0.37. More specifically, intellectual values was a significant positive predictor, β = 0.48, *t*(54) = 4.33, *p* < 0.001 (Hypothesis 3a). Smartphone usage time was a significant negative predictor, β =  − 0.30, *t*(54) =  − 2.64, *p* = 0.011 (Hypothesis 3b). Figure 2 shows the regression model and Fig. 3 shows the results as a 3D scatter plot with regression plane.

## Discussion

### Theoretical contributions and practical implications

Our findings add to the literature about note-taking’s effectiveness when learning with how-to videos. Unlike the extensive research that tested the note-taking effect under lab conditions, we focused on note-taking in a distance learning setting. More precisely, we transferred Peper, Shrager, and Mayer’s^10–12^ vintage experimental approach from the lab into a distance learning setting. As was the case taking this approach, we simply asked learners to take notes on a sheet of paper before providing them a short video (~ 12 min.). Furthermore, we aimed to analyze potential risk and protective factors for learning with how-to videos, such as smartphone usage time and intellectual values.

First, our findings demonstrate the effectiveness of our 12-min how-to video on plumbing, as it fostered the learners’ self-efficacy (Hypothesis 1). Second, we found that note-taking is an effective measure to foster both learning engagement (Hypothesis 2a) and learning outcomes (Hypothesis 2b). Encouraging learners take notes could simply be achieved by asking them to do so before the video. Obviously not everybody who was asked to take notes actually did so, but about 80% of the note-request receivers were compliant and reportedly took notes. From a practical perspective, asking learners to take notes is thus very advisable. It is only one little request right before the video, but it makes a significant difference in learning engagement and outcomes.

Furthermore, the note-taking effect on learning outcomes was mediated by learning engagement (Hypothesis 2c). This is in line with the previously discussed main function of note-taking. Note-taking is a generative activity^16^ involving various cognitive processes^9^ which benefit learning. The more engaged learners are in these processes, the better their performance in the posttest.

Moreover, in light of the known detrimental effects of digital distraction on learning and considering the enormous prevalence and excessive usage of smartphones these days, we assumed smartphone usage would be a risk factor for learning (Hypothesis 3a). We indeed detected smartphone usage time as a negative predictor on learning outcomes. Our sample’s mean smartphone usage time (~242 min.) was in line with previous studies^42–44,47^. It seems strongly advisable that learners avoid digital distractions, in particular through smartphones. Informing and educating learners about the negative associations between smartphone usage and learning could be a first small step. This advice appears even more useful in the light of research indicating that the mere presence of a smartphone can already occupy cognitive resources and thus hinder learning—even without any off-task behavior^65^.

Finally, we found that intellectual values contribute positively to learning outcomes. As mentioned previously, intellectual values reflect the degree to which a learner considers intellectual activity as worthwhile^50^. Short-term training interventions on how to foster intellectual values are rather few and far between^59^. After all, intellectual values are the product of a rather long development based on a sophisticated level of epistemological understanding^50^. Nevertheless, assessing learners’ intellectual values might help them (and their instructors) to become aware of the importance of valuing intellectual engagement. It might even spark some sort of self-reflection about learners’ willingness to invest mental effort and by doing so, help reduce digital distraction.

### Limitations and implications for future research

As was the case in all the classic note-taking studies discussed above^10–12^, we refrained from collecting and analyzing our participants’ notes. Hence, we had no objective data on learning processes such as ratings of note quality. However, we support the ecological validity advantages of not collecting the notes. Knowing that your notes will be collected could actually influence note-taking behavior and induce the bias of extra effort put in the notes. Furthermore, with Daumiller et al.’s^55^ scale on learning engagement, we already have a valid measure of the learning process. This instrument does not interfere with the learning process because it is employed afterwards. There are also no reasons for reservations about its validity because previous studies found high correlations with objective learning processes and outcomes^57,58^. Furthermore, this scale here proved able to distinguish between note-takers and non-note-takers (Hypothesis 2a) and served as a significant mediator of the note-taking effect on learning outcomes (Hypothesis 2c).

Our online field experiment relied on an asynchronous and unsupervised distance learning setting. Therefore, we cannot rule out that some participants might have used the internet or their notes during the test—although we asked them not to. Furthermore, the logged learning time does not enable us to differentiate between times spent on actual note-taking, to pause the video, or on off-task behavior. However, the positive correlation between learning time and learning outcomes and the low number of reported interruptions gave us no reason to assume any uncooperative behavior. For more objective data, future research might rely on eye-tracking or on-/off-screen recordings, however at the cost of ecological validity in a more lab-like scenario. Furthermore, more fine-grained measures for digital distraction, such as screen time and smartphone usage while watching the video, would help to analyze potential interactions with note-taking and learning outcomes. Moreover, it cannot be ruled out that the non-significant interaction term between note-taking and measurement time was due to the small sample size and a priori differences in self-efficacy.

A final potential limitation is our intervention’s short-term character. Admittedly, the video was only 12 minutes long. However, all the aforementioned classic studies on note-taking during video learning^10–12^ featured videos of similar length. Future studies might nevertheless include longer videos, and—more importantly—also implement delayed posttests. After all, previous studies showed deep processing’s importance for long term effectiveness, albeit under supervised and not under distance learning conditions.

## Conclusion

Summing up, our findings provide emphasis and empirical evidence on how beneficial it is for students to take notes while learning with how-to videos in a distance learning setting. Moreover, intellectual values can function as a protective factor for learning outcomes, whereas smartphone usage was exposed as a risk factor to learning from how-to videos. Consequently, learners should avoid digital distractions, especially by their smartphones.

## Data Availability

The dataset is available on OSF (https://osf.io/zx7r8).
